# Molecular sex determination in primates including non-invasive umbilical cord samples from neonates of three endangered species

**DOI:** 10.1007/s11033-026-12481-8

**Published:** 2026-07-25

**Authors:** Svatava Kubickova, Halina Cernohorska, Hana Hradska, Miluse Vozdova

**Affiliations:** Department of Genetics and Reproductive Biotechnologies, Veterinary Research Institute, Hudcova 70, Brno, 621 00 Czech Republic

**Keywords:** Molecular sexing, Sex determination, Non-invasive, Umbilical cord, *SRY* gene

## Abstract

**Background:**

Molecular sex determination is a valuable tool for an effective population management in captive bred endangered species. The molecular approaches are particularly valuable in species with limited sexual dimorphism. Moreover, the utilization of accessible non-invasive samples from newborn animals enables early sex determination beneficial for the animal population management and planning.

**Methods and results:**

A rapid molecular sex determination method using PCR with specific primers targeting the male-specific *SRY* locus and the control *c-myc* gene present in both sexes was developed. The method was validated using DNA samples from blood of adult males and females of Sumatran orangutan (*Pongo abelii*), western lowland gorilla (*Gorilla gorilla gorilla*), and silvery gibbon (*Hylobates moloch*). Then we applied the method to non-invasive samples from four newborns: two Sumatran orangutans, one western lowland gorilla, and one silvery gibbon. DNA of the newborns was isolated from umbilical cord material obtained *post partum* and/or hair. Additionally to the above mentioned species, the method was also successfully validated in adult males and females of chimpanzee (*Pan troglodytes*), pig-tailed macaque (*Macaca nemestrina*), Campbell’s monkey (*Cercopithecus campbelli*), mantled guereza (*Colobus guereza*), Geoffroy’s spider monkey (*Ateles geoffroyi*), and black-and-white ruffed lemur (*Varecia variegata*).

**Conclusions:**

We demonstrated that the umbilical cord material can be used for molecular sex determination in newborns and that the *c-myc* gene can serve as a control gene in molecular sex determination, providing an alternative to other commonly used methods.

**Supplementary Information:**

The online version contains supplementary material available at 10.1007/s11033-026-12481-8.

## Introduction

In captive bred animals, early sex determination is important for proper social grouping, long-term planning and breeding management. Molecular sexing is also a valuable tool in conservation and wildlife studies because it enables accurate sex determination, especially in species with limited sexual dimorphism. Reliable sex identification can support population monitoring, demographic analyses, and the subsequent development of effective conservation and management strategies for threatened and endangered species. The molecular approach is beneficial especially because it enables utilization of non-invasive samples such as hair [1–3], feces [4, 5], feathers [6] and saliva [7]. Despite potential limitations, including reduced DNA yield or molecular degradation, analysis of non-invasive samples remains a highly effective tool eliminating the need to capture, handle, or immobilize the animals [8, 9]. That is especially valuable for rare or endangered taxa.

Molecular sex determination has been used in a wide range of wild and captive bred species, as, e.g., bears, goral, sloth, rhinoceros, hyenas and birds [3, 6, 8–11]. In this study, we focused on primates, where several methodological approaches have been employed for molecular sex determination. These include either PCR amplification of homologous X- and Y-specific regions that differ in the fragment length, such as the amelogenin (AMELX/AMELY) loci [4, 12, 13] and the zinc-finger protein ZFX/ZFY genes [12, 14]. However, the AMELX/AMELY system was not proved suitable for sex identification in some primate species [5]. Villesen and Fredsted [12] developed an alternative method based on the amplification of short UTX/UTY fragments, which exhibit larger X–Y size differences and can thus be more easily resolved on the agarose gel than the AMELX/AMELY system [15]. Also a method based on the amplification of the male-specific *SRY* (sex-determining region) gene and a control marker (AMELX or UTX/UTY) that amplifies in both sexes was reported [16–19]. In some studies, cytochrome b and *c-myc* gene have also been used as internal control for molecular sexing [3, 20]. The functional domains of the *c-myc* gene exhibit high evolutionary conservation [21], and the gene thus seems to be suitable as the control marker for the sex determination in a range of mammalian species.

This study aimed to introduce an alternative molecular sex determination method in primates and evaluate its practical applicability for umbilical cord and hair from newborns of three endangered primate species: the western lowland gorilla (*Gorilla gorilla gorilla*, Hominidae), the Sumatran orangutan (*Pongo abelii*, Hominidae) and the silvery gibbon (*Hylobates moloch*, Hylobatidae). The umbilical cord cells originate from the fetus (Can and Karahuseyinoglu 2007), and the umbilical cord tissue thus represents a non-invasive and ethically acceptable source of DNA in endangered species. Its use enables accurate sex identification without the need for additional handling or sampling of neonates, thereby minimizing stress to the animals and possible danger to their keepers. To assess the general applicability of the primers designed for in this study to a broader range of primates, we also successfully tested the method in adult males and females of additional six primate species from both Haplorhini and Strepsirrhini: the chimpanzee (*Pan troglodytes*, Hominidae), the pig-tailed macaque (*Macaca nemestrina*, Cercopithecidae), the Campbell’s monkey (*Cercopithecus campbelli*, Cercopithecidae), the mantled guereza (*Colobus guereza*, Cercopithecidae), the Geoffroy’s spider monkey *(Ateles geoffroyi*, Atelidae), and the black-and-white ruffed lemur (*Varecia variegata*, Lemuridae).

## Materials and methods

### Samples

Standard blood samples were collected from one male and one female of each of the nine analysed primate species (western lowland gorilla, Sumatran orangutan, silvery gibbon, chimpanzee, pig-tailed macaque, Campbell’s mona monkey, mantled guereza, Geoffroy’s spider monkey, and black-and-white ruffed lemur). All animals were kept in continuous group housing with full-contact conditions in the Prague Zoo and Zoo Dvůr Králové nad Labem (Czech Republic). The zoo veterinarians retrieved the blood samples during routine preventive examinations or other clinical procedures, and a spared aliquot of up to 0.5 ml was provided to be stored in our laboratory for future molecular analyses, as the one currently reported.

For the non-invasive sex determination in newborns, umbilical cord samples were collected from a western lowland gorilla (cord blood) and from two Sumatran orangutans (cord tissue) by animal keepers 3 to 5 days after the birth of the animals while cleaning the housing facility. They also collected the hair sample from the young silvery gibbon.

### DNA isolation, PCR, cloning and sequencing

A total of 200 µl of peripheral blood and 30 mg of umbilical cord tissue were used for DNA isolation with the QIAamp DNA Blood Mini Kit and QIAamp DNA Mini Kit (Qiagen, Hilden, Germany), respectively. The DNA from hair bulbs was obtained using the previously described procedure [19]. Briefly, approximately 15 hair bulbs were immersed in 5% Chelex (Bio-Rad, Hercules, USA), incubated at 56°C overnight, boiled for 8 min, and centrifuged for 5 min at 10,000 g. The resulting supernatant was used for PCR. DNA isolated from a total of 22 individuals (i.e., 9 adult males and 9 females and 4 newborns of the primate species listed above) was used for the PCR amplification of the male-specific region (*SRY*) and the control *c-myc* gene fragment. The male-specific primers were designed based on the *SRY* sequence of the Bornean orangutan (*Pongo pygmaeus*) available in the NCBI database (X86383.1). The *SRY* region amplification was performed using the primer pair 5’-GAGTGAAGCGACCCATGAACG-3’ and 5’-TGTGCCTCCTGGAAGAATGGC-3’, yielding a 166 bp-long fragment. Amplification of the control *c*-*myc* gene (NCBI sequence X97040) was carried out using the primer pair 5’-ACCAGTTGGAGATGGTGACCGAGC-3’ and 5’-CGTTGAGCGGGTAGGGGAAGACC-3’ (Vozdova et al. 2019), producing a 313 bp fragment. All PCR reactions were performed using the Hot Start Combi PPP Master Mix (Top-Bio, Vestec, Czech Republic) according to the manufacturer’s instructions, with an annealing temperature of 62 °C. Cycling parameters were: 95 °C for 4 min for initial denaturation, 36 cycles at 95 °C for 45 s, 62 °C for 35 s, and 72 °C for 60 s, with a 5 min final extension at 72 °C. The PCR products derived from the *SRY* region of the male western lowland gorilla, Sumatran orangutan, and silvery gibbon were subsequently cloned into a pDrive Cloning Vector (Qiagen), sequenced, and the resulting sequences were analysed for similarity using BLASTN (https://blast.ncbi.nlm.nih.gov) and ClustalW (https://www.genome.jp/tools-bin/clustalw) software.

## Results

**Fig. 1:**
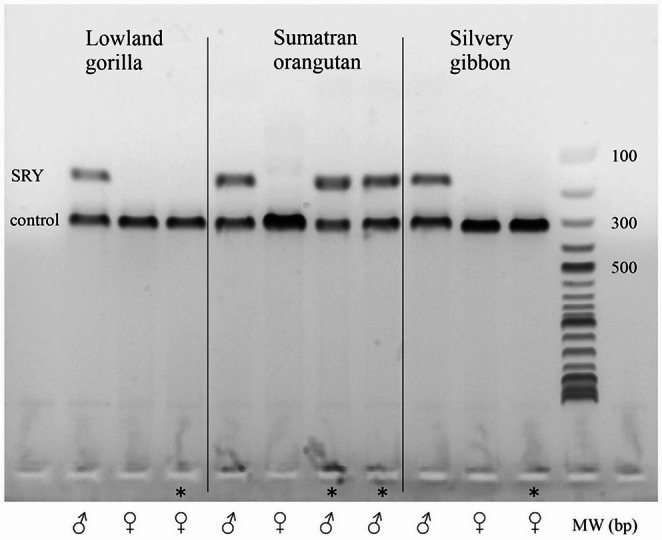
Electrophoretic gel documenting results of the molecular sex determination in the western lowland gorilla, Sumatran orangutan and silvery gibbon using PCR amplification of the *SRY* region and the autosomal control gene (*c-myc*). Asterisks indicate results obtained from non-invasive samples collected from newborn animals of previously unknown sex. MW – molecular weight marker

Initially, we validated the designed primers by PCR amplification of the targeted sequences in male and female samples from adult western lowland gorilla, Sumatran orangutan and silvery gibbon. Using gel electrophoresis, we proved that the control *c-myc* gene sequence was amplified in both sexes, whereas the amplification of the *SRY* gene fragment was specific to males, resulting thus to two distinct bands (166 bp and 313 bp) on the electrophoretic gel in male samples and one band (313 bp) in females (Fig. 1).

The sequences of the cloned *SRY* gene fragment obtained from western lowland gorilla, Sumatran orangutan, and silvery gibbon in this study were compared using BLASTN software, and the results revealed high similarity (99.8%, 100%, and 99.8%, respectively) with the Bornean orangutan (*Pongo pygmaeus*) NCBI sequence X86383.1 used for the primer design. The ClustalW alignment of the *SRY* fragment sequences obtained in this study for the male western lowland gorilla, Sumatran orangutan and silvery gibbon with *SRY* gene sequences available for these species in the NCBI database showed high similarity with only one nucleotide substitution in western lowland gorilla, and one substitution in silvery gibbon. The results are presented in Online Resource 1 (ESM 1).

Subsequently, we utilised the method for molecular sex determination in newborn animals of the three mentioned species using DNA isolated from the non-invasive samples (umbilical cord blood in western lowland gorilla, umbilical cord tissue in two Sumatran orangutans and hair in silvery gibbon). The results showed that the tested newborn western lowland gorilla and silvery gibbon were females, whereas the two newborn Sumatran orangutans were males. The results are displayed in Fig. 1. The sex of all young individuals was later confirmed by visual observation of the animals.

Finally, we tested the possibility of utilisation of the method in the six additional primate species (chimpanzee, pig-tailed macaque, Campbell’s mona monkey, mantled guereza, Geoffroy’s spider monkey, and black-and-white ruffed lemur). As expected, two bands were observed on the electrophoretic gel in males and one band in females in all analysed species.

## Discussion

Reliable sex determination is an essential tool in conservation, zoological management and captive breeding programmes, population monitoring, and behavioural studies [22, 23]. In the present study in primates, we developed and validated a simple PCR-based assay targeting the male-specific *SRY* gene together with the autosomal *c-myc* gene as an internal amplification control. The inclusion of an internal control gene is particularly important when analysing samples of variable quality, as it allows discrimination between female samples and false-negative results caused by poor DNA quality [10, 20]. In this study, the method successfully discriminated between male and female individuals in all tested species and sample types, showing its potential applicability across a broad range of primates including both Haplorhini and Strepsirrhini.

An important outcome of this work is the successful application of the method to DNA obtained from non-invasive or minimally invasive samples collected from newborn individuals. Accurate sex determination of neonates can be challenging because external sexual characteristics may not be sufficiently developed or the social behaviour of the animals prevents reliable visual identification. In silvery gibbon, e.g., there is no apparent sexual dimorphism even in adults (https://animaldiversity.org/accounts/Hylobates_moloch/), and the baby is kept close to the mother for the first year of life (https://neprimateconservancy.org/silvery-gibbon/). In zoo populations, early determination of sex can facilitate animal management, pedigree records, and demographic planning. The successful molecular sex determination from non-invasive samples demonstrates the robustness of the assay and its suitability for situations where collection of standard blood samples is impractical or undesirable.

As mentioned, we also demonstrated that this method can be used for sex determination from umbilical cord samples. As far as we know, this is the first study reporting utilization of the umbilical cord material for sex determination in non-human primates. The umbilical cord serves as a vital connection between the fetus and placenta, and contains cells that originated from the fetus [24]. In captive bred species, the umbilical cord represents a unique biological resource, that can usually be obtained after delivery from the animal facilities without the need for invasive intervention. This material provides a valuable source of biological material for genetic analyses, eliminating the need to directly access the newborn animals, which can be stressful for the mother and newborn and potentially hazardous for the staff. In some cases, access to the umbilical cord material may be delayed. It was also the case of this study, where we obtained the placenta with the adjacent umbilical cord material of the newborn gorilla and orangutans three to five days after delivery. At this time point the material was partly desiccated. DNA from such samples can be degraded and longer fragments may be poorly amplified using PCR [25]. For this reason, we selected primers that generated shorter DNA fragments (166 bp for *SRY* and 313 bp for the control *c-myc* gene) to accommodate the potential partial DNA degradation in the dry samples.

Several molecular methods for sex determination have been developed for primates, e.g. [13, 17, 19] . Nevertheless, their applicability may be limited across different species because sequence variation can affect primer binding and amplification efficiency [5]. In this study, sequence analysis of the amplified *SRY* fragment and alignment with species-specific sequences already available in the NCBI database revealed only minor single nucleotide differences. The observed conservation of the targeted *SRY* region, together with the successful application of the assay in representatives of both Haplorhini and Strepsirrhini, suggests that the method may be applicable to additional primate species. However, its performance should be validated individually for each species before routine application.

## Conclusion

In summary, our results show that that there is a high sequence similarity in the *SRY* gene fragment among the analysed species, and that the autosomal *c-myc* gene can be used as a control marker for molecular sexing across diverse mammalian taxa. We also proved that umbilical cord could serve as a non-invasive source material for molecular analysis in newborn captive bred animals, especially in endangered species, where any extensive handling of the animals is undesirable.

## Supplementary Information

Below is the link to the electronic supplementary material.

Supplementary Material 1

## Data Availability

All data are included in this article; no additional datasets were generated or analysed during the current study.
