# Supplementary material for: Molecular sex determination in primates including non-invasive umbilical cord samples from neonates of three endangered species
Source: Mol Biol Rep. 2026 Jul 25;53(1):1265. doi: 10.1007/s11033-026-12481-8 (PMC13401576; doi:10.1007/s11033-026-12481-8)
Supplement: Supplementary file 1 — Supplementary Material 1 [file 11033_2026_12481_MOESM1_ESM.pdf]

Supplementary material

Online Resource 1: Comparison of the SRY sequences (166 bp) obtained in this study (underlined) in the western lowland gorilla, Sumatran orangutan and silvery gibbon with those accessible for these species in the NCBI Genbank (accession numbers are given next to each species) The comparison was performed using ClustalW software. The nucleotide differences are marked in red and blue.

```
Pongo pygmaeus X86383          GAGTGAAGCGACCCATGAACGCATTCATCGTGTGGTCTCGCGATCAGAGG
Pongo abelii                   GAGTGAAGCGACCCATGAACGCATTCATCGTGTGGTCTCGCGATCAGAGG
Gorilla gorilla X86382         GAGTGAAGCGACCCATGAACGCATTCATCGTGTGGTCTCGCGATCAGAGG
Gorilla gorilla                GAGTGAAGCGACCCATGAACGCATTCATCGTGTGGTCTCGCGATCAGAGG
Hylobates moloch XM_032756524  GAGTGAAGCGACCCATGAACGCATTCATCGTGTGGTCTCGCGATCAGAGG
Hylobates moloch               GAGTGAAGCGACCCATGAACGCATTCATCGTGTGGTCTCGCGATCAGAGG
                               **************************************************

Pongo pygmaeus X86383          CGCAAGATGGCTCTAGAGAATCCCAAAATGCGAAACTCAGAGATCAGCAA
Pongo abelii                   CGCAAGATGGCTCTAGAGAATCCCAAAATGCGAAACTCAGAGATCAGCAA
Gorilla gorilla X86382         CGCAAGATGGCTCTAGAGAATCCCAGAATGCGAAACTCAGAGATCAGCAA
Gorilla gorilla                CGCAAGATGGCTCTAGAGAATCCCAGAATGCGAAACTCAGAGATCAGCAA
Hylobates moloch XM_032756524  CGCAAGATGGCTCTAGAGAATCCCAAAATGCGAAACTCAGAAATCAGCAA
Hylobates moloch               CGCAAGATGGCTCTAGAGAATCCCAAAATGCGAAACTCAGAAATCAGCAA
                               ************************* *************** ********

Pongo pygmaeus X86383          GCAGCTGGGATACCAGTGGAAAATGCTTACTGAAGCCGAAAAATGGCCAT
Pongo abelii                   GCAGCTGGGATACCAGTGGAAAATGCTTACTGAAGCCGAAAAATGGCCAT
Gorilla gorilla X86382         GCAGCTGGGATACCAGTGGAAAATGCTTACTGAAGCCGAAAAATGGCCAT
Gorilla gorilla                GCAGCTGGGATACCAGTGGAAAATGCTTACTGAAGCCGAAAAATGGCCAT
Hylobates moloch  M_032756524  GCAGCTGGGATACCAGTGGAAAATGCTTACTGAAGCCGAAAAATGGCCAT
Hylobates moloch               GCAGCTGGGATACCAGTGGAAAATGCTTACTGAAGCCGAAAAATGGCCAT
                               **************************************************

Pongo pygmaeus X86383          TCTTCCAGGAGGCACA
Pongo abelii                   TCTTCCAGGAGGCACA
Gorilla gorilla X86382         TCTTCCAGGAGGCACA
Gorilla gorilla                TCTTCCAGGAGGCACA
Hylobates moloch XM_032756524  TCTTCCAGGAGGCACA
Hylobates moloch               TCTTCCAGGAGGCACA
                               ****************
```
